# Post-operative pulmonary complications after thoracic and upper abdominal procedures at referral hospitals in Amhara region, Ethiopia: a multi-center study

**DOI:** 10.3389/fsurg.2023.1177647

**Published:** 2023-05-15

**Authors:** Biruk Adie Admass, Birhanu Yilma Ego, Hailu Yimer Tawye, Seid Adem Ahmed

**Affiliations:** ^1^ Department of Anesthesia, College of medicine and health sciences, University of Gondar, Gondar; ^2^ Department of Anesthesia, College of medicine and health sciences, Arba Minch University, Arba Minch, Ethiopia

**Keywords:** pulmonary complication, thoracic surgery, upper abdominal surgery, respiratory failure, anesthetics

## Abstract

**Background:**

Thoraco-abdominal surgery cuts through muscle, disrupting the normal structure and function of the respiratory muscles, resulting in lower lung volumes and a higher risk of developing post-operative pulmonary complications (PPC). PPC remains an important cause of post-operative morbidity and mortality and impacts the long-term outcomes of patients after hospital discharge. This study was aimed at determining the incidence and factors associated with postoperative pulmonary complications among patients who underwent thoracic and upper abdominal surgery in the Amhara region of Ethiopia.

**Methods:**

A multi-center follow-up study was conducted from April 1, 2022, to June 30, 2022, at comprehensive specialized hospitals in Amhara regional state, northwest Ethiopia. 424 patients were consecutively included in this study, with a response rate of 100%. A chart review and patient interview were used to collect data. A logistic regression analysis was performed to assess the strength of the association of independent variables with postoperative pulmonary complications.The crude odds ratio (COR) and adjusted odds ratio (AOR) with the corresponding 95% confidence interval were computed. Variables with a *p*-value of <0.05 were considered statistically significant predictors of the outcome variable.

**Results:**

The incidence of postoperative pulmonary complication was 24.5%. Emergency procedures, preoperative SpO_2_ < 94%, duration of surgery >2 h, patients with a nasogastric tube, intraoperative blood loss >500 ml and post-operative albumin <3.5 g/dl were factors associated with pulmonary complications. The most common complications were pneumonia (9.9%) followed by respiratory infection (4.2%).

**Conclusion:**

The incidence of postoperative pulmonary complication after thoracic and upper abdominal surgery remains high. Preoperative SpO_2_, duration of surgery, patients having a nasogastric tube, intraoperative blood loss and post-operative albumin were factors associated with post-operative pulmonary complications.

## 1. Introduction

Surgical procedures are predicted to account for 11% of total global disease burden and 25 million disability-adjusted life years (DALYs) in Africa, the region with the highest concentration of surgical DALYs (38/1,000 population) (1). Surgery and trauma patients typically experience a sudden and significant systemic insult that commonly results in the decompensation of subclinical illnesses and creates potentially fatal effects (2). A high mortality rate in surgical units was caused by the fact that the majority of post-surgical problems were either surgical or anesthetic care-related (3).

The most common cause of morbidity following surgery is still post-operative problems involving the respiratory system (4). Postoperative pulmonary complications (PPC) are adverse changes to the respiratory system that occur after surgery and can adversely affect the patient's clinical outcome (5). A pulmonary abnormality that develops following surgery and leads to clinically significant disease or dysfunction, adversely influencing the clinical course of the initial condition, is referred to as a post-operative pulmonary complication (PPC) (6). There is no well recognized definition of PPC. Many literatures used the definition of PPC, which typically includes acute respiratory failure, weaning failure, pneumonia, atelectasis, bronchospasm, worsening of chronic obstructive pulmonary disease, pneumothorax, pleural effusion, and different kinds of upper airway obstruction (5, 7–9).

Postoperative pulmonary complications (PPC) are the leading causes of morbidity and mortality, specifically within the first postoperative week (7, 10). The incidence of PPC is inversely related to the distance of the surgical incision from the diaphragm, making patients undergoing abdominal and thoracic surgery the most vulnerable (11). Postoperative pulmonary complications are more common in thoracic and abdominal surgeries, with an incidence varying from 12% to 70% (12). The incidence is mainly related to the patient population, the type of surgery, and the presence of contributing factors (13). It has been estimated that more than 1 million PPCs occur annually in the United States, resulting in 46,200 related deaths and 4.8 million additional hospitalization days (14).

Postoperative pulmonary complications after thoracic and upper abdominal surgery may have a significant clinical and economic impact, as they are associated with a longer length of hospital stay and costs, a higher frequency of intensive care unit (ICU) admissions, and a higher number of hospital deaths (13, 15–17). PPC after thoracic and upper abdominal surgery is significantly more common in patients with cardiopulmonary disturbances and coexisting diseases (12, 18). Preoperative evaluation and pulmonary risk stratification are important parts of primary care (19). Prevention of PPC requires a multidisciplinary approach that includes optimization of co-morbidities, greater utilization of regional analgesia, balancing intravenous fluid therapy, gastric drainage, vigilance monitoring, chest physiotherapy, lung expansion maneuvers, early mobilization, and early oral feeding in the postoperative period (13, 20).

PPC is still a major problem, and there are a limited number of studies on the incidence and factors associated with post-operative pulmonary complications after thoracic and upper abdominal surgery among surgical patients in Ethiopia. Therefore, this study was aimed at determining the incidence and factors associated with postoperative pulmonary complication in patients who underwent thoracic and upper abdominal surgery at comprehensive specialized hospitals in the Amhara region of Ethiopia.

## 2. Methods and materials

### 2.1. Study design and setting

A prospective multi-center follow-up study was conducted from April 1, 2022, to June 30, 2022, at comprehensive specialized hospitals in Amhara national regional state, northwest Ethiopia. This research was carried out at four comprehensive specialty hospitals in Ethiopia's Amhara region. The research was conducted at the University of Gondar, Tibebe-Ghion, Debre-Markos, and Felege-Hiwot comprehensive specialized hospitals.

The University of Gondar comprehensive specialized hospital is located in the Amhara region's Gondar town. The hospital has nearly 400 beds and eleven operating rooms, and it serves as a referral center for numerous district hospitals. It provides service for more than seven million people.

Tibebe-Ghion and Felege-Hiwot comprehensive specialty hospitals are located in Bahir-Dar City, the region's capital, and have eleven and four operating rooms, respectively. Debre-Markos comprehensive specialty hospital is 265 kilometers from Bahir-Dar city. This referral hospital has four operating rooms and serves more than five million people.

All elective and emergency adult patients who had thoracic and upper abdominal surgery during the study period were included in the study. Intraoperative death, preoperatively intubated patients, patients who refuse to give consent and are unable to communicate, and pregnant women were excluded from the study.

### 2.2. Operational definition

According to European Perioperative Clinical Outcome (EPCO) diagnostic criteria and definition, a postoperative pulmonary complication is the occurrence of one or more of the following during the first postoperative week: respiratory infection, respiratory failure, pleural effusion, atelectasis, pneumothorax, bronchospasm, aspiration pneumonitis, pneumonia, acute respiratory distress syndrome, pulmonary edema, and tracheobronchitis (21).

### 2.3. Sample size calculation

The sample size was calculated using the single population proportion formula. Since there is no similar previous study in Ethiopia, the assumption of a 50% proportion, a 5% margin of error, and a 95% confidence interval was used.

$$n=\frac{\;p(1-p){\left(z\alpha /2\right)}^{2}}{d^{2}}$$

$$n=\frac{0.5\times [1-0.5]{\left[1.96\right]}^{2}}{{\left[0.05\right]}^{2}}$$

n=384.

where, *n*: sample size, *p*: proportion, d: absolute precision. 424 patients were included in the study overall after adding 10% of the non-response rate.

### 2.4. Sampling technique

Patients who underwent thoracic and upper abdominal surgery were consecutively included until the required sample size was reached. Patients who were admitted preoperatively to the selected hospitals and underwent elective and emergency thoracic and upper abdominal surgery were evaluated for PPC. A review of the previous surgeries per year on the log book showed that there were 1,728 surgeries, and the number of operations per three months was 432. Therefore, during the study period, 432 patients were estimated to have undergone thoracic and upper abdominal surgery at four comprehensive specialized hospitals in the Amhara region.

**Figure 1:**
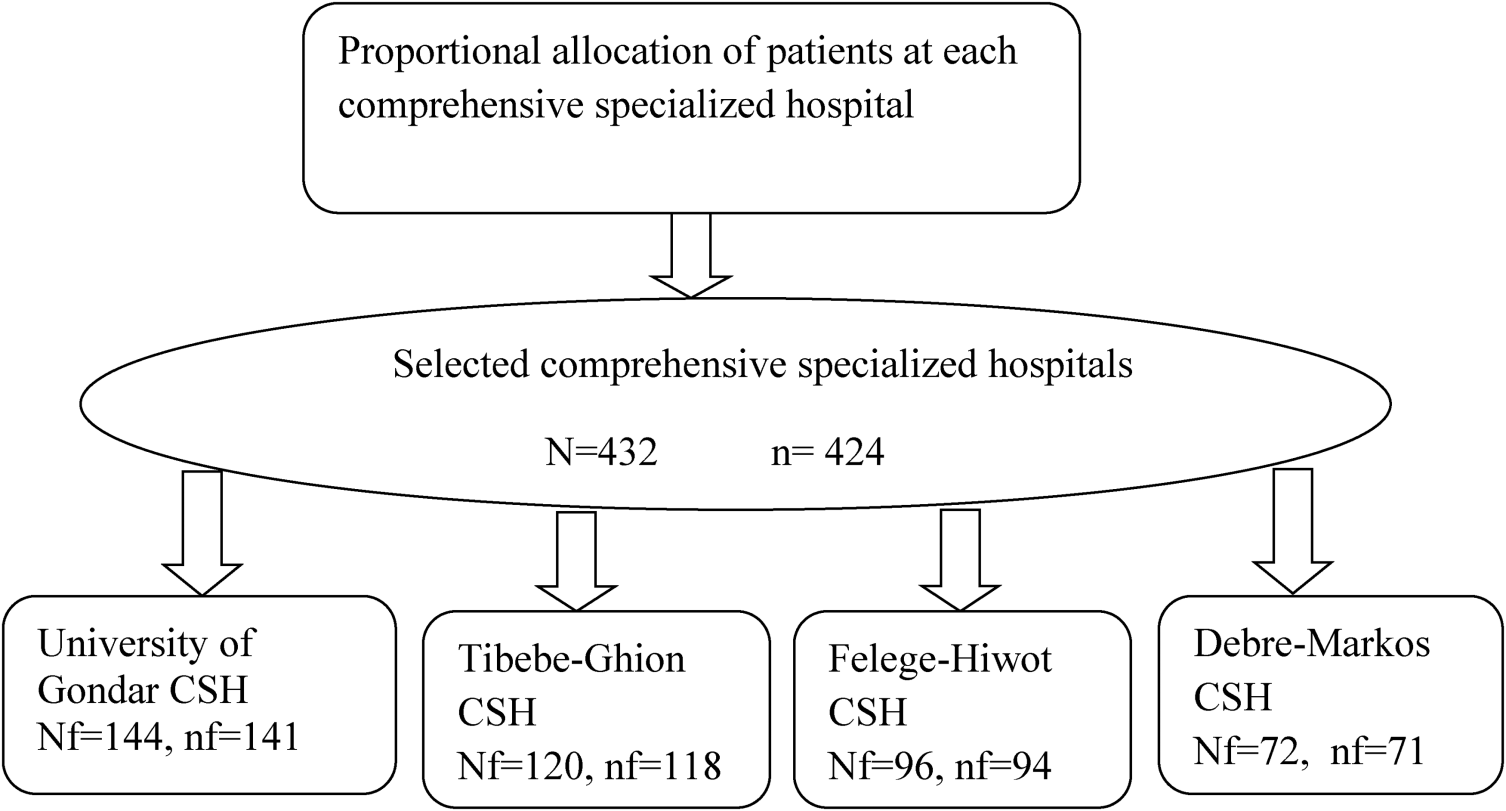
Proportional allocation and enrollment of patients who underwent thoracic and upper abdominal surgery at comprehensive specialized hospitals in the Amhara region, Ethiopia. CSH, Comprehensive specialized hospital.

The sample size for each hospital was calculated using nf = (Nf/N) n, where nf is the allocated sample size of each hospital, N is the total population of the comprehensive specialized hospitals, n is the calculated sample size, and Nf is the size of each hospital's source population (Figure 1).

### 2.5. Data collection instrument and procedure

The data were collected through interviews and chart reviews. Two trained data collectors were assigned at each center to collect the data, consisting of demographic characteristics and perioperative surgical and anesthetic-related variables of the patient. Postoperative pulmonary complications were assessed in the patient's chart. Physician and nursing notes, order sheets, anesthesia records, operative reports, and diagnostic studies were reviewed.

Patients were considered as having post-operative pulmonary complication (PPC) when one or more of the following: pneumonia, tracheo-bronchitis, atelectasis, respiratory failure, respiratory infection, bronchospasm, aspiration pneumonitis, pulmonary edema, pneumothorax, and pleural effusion were occurred during the first postoperative week. Patients were monitored daily, and follow-up started on the day of the surgery and ended within 7 days postoperatively. The data collectors report to the physicians when they observe a pulmonary complication.

### 2.6. Data quality control

To ensure the quality of the data, a pre-test was conducted on 22 (5%) of the patients who were not included in the main study at the University of Gondar comprehensive specialized hospital. Data collectors and supervisors were trained for data collection procedures. The supervisors monitored the data collector and checked for completeness every day following data collection. The collected data was reviewed for completeness, accuracy, and clarity.

### 2.7. Data processing and analysis

Epi-Data version 4.6 was used to enter the data and SPSS version 20 for analysis. Socio-demographic, surgical, and anesthetic variables of the patients were analyzed and presented in tables. Model fitness was checked by the Hosmer-Lemeshow test. Bivariable and multivariable logistic regression analyses were performed to determine the association between dependent and independent variables. Variables with a *p*-value of less than 0.2 in binary logistic regression were transferred into the multivariable logistic regression model to analyze the factors associated with postoperative pulmonary complications after thoracic and upper abdominal surgery. Both the crude odds ratio (COR) and the adjusted odds ratio (AOR) with the corresponding 95% confidence interval (CI) were calculated to show the strength of the association of risk factors with post-operative pulmonary complications. Variables with a *p*-value of less than 0.05 in the multivariate logistic regression were considered statistically significant predictors of pulmonary complications after upper abdominal and thoracic surgery.

### 2.8. Ethical consideration

The research and ethical review committee of the college of medicine and health sciences at the University of Gondar granted its approval for the study, which was then carried out. Each hospital's medical director provided a letter of permission for the study to be carried out. The patient was told about the study, and each participant gave written informed consent to the data collectors. Avoiding using personal identifiers allowed for the preservation of confidentiality throughout the entire study. The patients' participation in the study was voluntary, and those who did not want to participate or who changed their minds about participating at any time were allowed to do so.

## 3. Results

### 3.1. Socio-demographic and clinical characteristics of the patients

A total of 424 patients who underwent thoracic and upper abdominal surgery were included in this study, with a response rate of 100%. The majority of patients, 256 (60.4%), were males with a median age of 37 years. Most of the patients, 320 (75.5%), were classified as ASA I. Only 24 (5.7%) patients were smokers, and 35 (8.3%) were alcohol drinkers. 258 (60.8%) procedures were emergency operations. The majority of the patients', 364 (85.8%), preoperative hemoglobin was >10 g/dl (Table 1).

**Table 1:** Socio-demographic and clinical characteristics of the patients who underwent thoracic and upper abdominal surgery at comprehensive and specialized hospitals in Amhara region, Ethiopia.

Variables	Category	Total (*n*, %)	PPC	
			Yes (*n*, %)	No (*n*, %)
Age (year)	≥65	36 (8.5)	14 (38.9)	22 (61.1)
Sex	Male	256 (60.4)	64 (25.0)	192 (75.0)
BMI	<18.5	50 (11.8)	26 (52)	24 (48)
	18.5–24.9	329 (77.6)	66 (20.1)	263 (79.9)
	25–30	39 (9.2)	11 (28.2)	28 (71.8)
	>30	6 (1.4)	1 (16.7)	5 (83.3)
ASA	I	320 (75.5)	73 (22.8)	247 (77.2)
	II	96 (22.6)	26 (27.1)	70 (72.9)
	III	8 (1.9)	5 (62.5)	3 (37.5)
Preoperative hemoglobin (g/dl)	≤10	60 (14.2)	31 (51.7)	29 (48.3)
Category of surgery	Emergency	258 (60.8)	77 (29.8)	181 (70.2)
Smoking status	Yes	24 (5.7)	9 (37.5)	15 (62.5)
Co-morbidities	Neoplasm	13 (3.1)	4 (30.8)	9 (69.2)
	HIV	24 (5.7)	7 (29.2)	17 (70.8)
	Hypertension	23 (5.4)	4 (17.4)	19 (82.6)
	DM	15 (3.5)	4 (26.7)	11 (73.3)
	COPD/asthma	8 (1.9)	3 (37.5)	5 (62.5)
	None	341 (80.4)	82 (24)	259 (76)
Preoperative antibiotic use	No	138 (32.5)	34 (24.6)	104 (75.4)
Preoperative Spo_2_ (%)	<94	47 (11.1)	26 (55.3)	21 (44.7)
Respiratory infection	Yes	14 (3.3)	6 (42.9)	8 (57.1)
Alcohol drinker	Yes	35 (8.3)	9 (25.7)	26 (74.3)

BMI, body mass index; ASA, American society of anesthesiologist; Preoperative Spo_2_: oxy-hemoglobin saturation; HIV, human immunodeficiency virus; DM, diabetes mellitus; COPD, chronic obstructive pulmonary disease; PPC, postoperative pulmonary complication.

### 3.2. Surgical and anesthetic related characteristics of patients

The majority of patients underwent upper abdominal surgery, which accounts for 96.9% of operations, and about 23.8% of patients had a nasogastric tube *in situ*. About 66.3% of patients had a blood loss of less than 500 ml. Only 3 (0.7%) patients underwent laparoscopic operations, and the majority of the patients received opioid. The majority of patients’ (71.7%) postoperative albumin level was greater than 3.5 g/dl (Table 2).

**Table 2:** Surgical and anesthetic related characteristics of patients who underwent thoracic and upper abdominal surgery at comprehensive specialized hospitals in Amhara region, Ethiopia.

Variables	Category	Total (*n*, %)	PPC	
			Yes (*n*, %)	No (*n*, %)
Duration of surgery (hr)	≤2	125 (29.5)	11 (8.8)	114 (91.2)
	2–3	184 (43.4)	41 (22.3)	143 (77.7)
	>3	115 (27.1)	52 (45.2)	63 (54.8)
Surgery site	Thoracic	13 (3.1)	6 (46.2)	7 (53.8)
	Upper abdominal	411 (96.9)	98 (23.8)	313 (76.2)
Nasogastric tube *in situ*	Yes	101 (23.8)	51 (50.5)	50 (49.5)
Intraoperative blood loss	>500	143 (33.7)	63 (44.1)	80 (55.9)
Induction agent	Ketamine	192 (45.3)	47 (24.5)	145 (75.5)
	Propofol	39 (9.2)	7 (17.9)	32 (82.1)
	Thiopental	7 (1.7)	1 (14.3)	6 (85.7)
	Ketofol	186 (43.9)	49 (26.3)	137 (73.7)
Muscle relaxant	Vecuronium	35 (8.3)	10 (28.6)	25 (71.4)
	sux + Vecuronium	388 (91.5)	94 (24.2)	294 (75.8)
	Sux + Pancuronium	1 (0.2)	0	1 (100.0)
Maintenance anesthesia	Halothane	85 (20.0)	25 (29.4)	60 (70.6)
	Isoflurane	339 (80.0)	79 (23.3)	260 (76.7)
Type of operation	Open surgery	421 (99.3)	104 (24.7)	317 (75.3)
	Laparoscopy	3 (0.7)	0	3 (100.0)
Type of analgesia	Opioid	357 (84.2)	94 (26.3)	263 (73.7)
	Opioid + NSAID	42 (9.9)	10 (23.8)	32 (76.2)
	Multimodal analgesia	25 (5.9)	0	25 (100.0)
Postoperative albumin (g/dl)	<3.5	120 (28.3)	68 (56.7)	52 (43.3)
Fasting blood glucose (mmol/L)	≤6.1	91 (21.5)	33 (36.3)	58 (63.7)

NSAID, non-steroidal anti inflammatory drug; PPC, postoperative pulmonary complication; Sux, suxamethonium.

### 3.3. Incidence of post-operative pulmonary complications after thoracic and upper abdominal surgery

**Figure 2:**
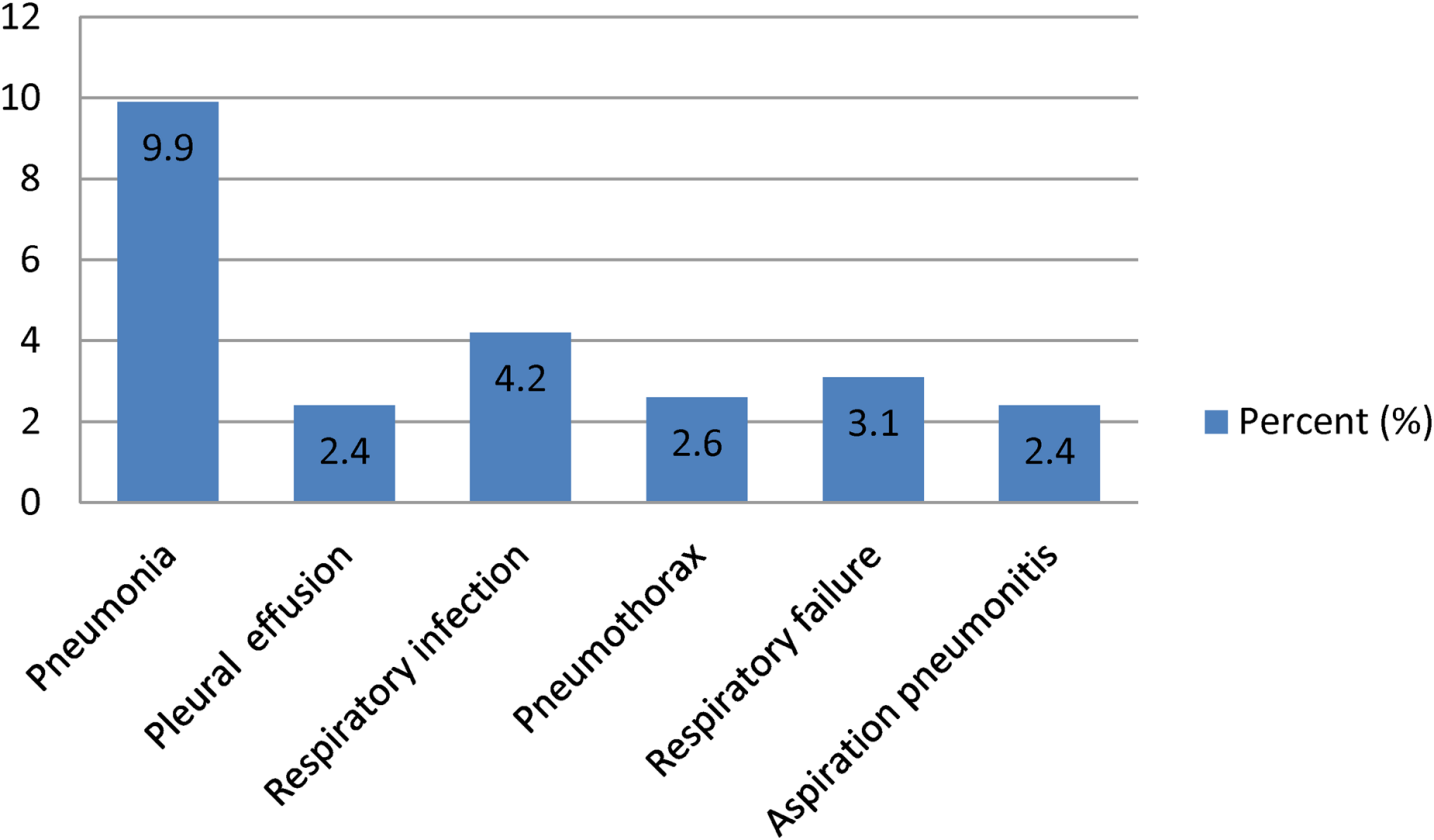
Types of post-operative pulmonary complications.

**Figure 3:**
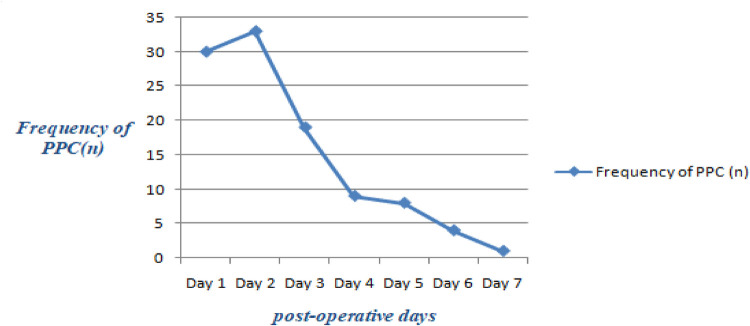
The frequency of post-operative pulmonary complications in the respective post-operative days of a week.

The overall incidence of postoperative pulmonary complications after thoracic and upper abdominal surgery was 104 (24.5%) (95% CI: 20.5–28.5). The most common complications were pneumonia (42, 9.9%), followed by respiratory infection (18, 4.2%), and respiratory failure (13, 3.1%) (Figure 2). The majority of the complications occurred within the first three days of the post-operative period (Figure 3). From the total PPCs that occurred, 95 patients had a single PPC, seven patients had two PPC, and two patients had three postoperative pulmonary complications.

### 3.4. Factors associated with post-operative pulmonary complication after thoracic and upper abdominal surgery

In the bi-variable logistic regression analysis, variables like age, ASA, preoperative anemia, category of surgery, preoperative Spo_2_, duration of surgery, NG tube *in situ*, intraoperative blood loss, post-operative albumin, and post-operative fasting blood glucose had a *p*-value of <0.2 and were considered as potential factors associated with post-operative pulmonary complications (Table 3).

**Table 3:** Bi-variable logistic regression analysis of factors associated with post-operative pulmonary complications after thoracic and upper abdominal surgery at comprehensive specialized hospitals in Amhara region, Ethiopia.

		PPC		Odds ratio	
Variables	Category	Yes (*n*, %)	No (*n*, %)	COR (95% CI)	*p*-value
Age (years)	<65	90 (23.2)	298 (76.8)	1	0.040
	≥65	14 (38.9)	22 (61.1)	2.107 (1.036–4.287)	
ASA	I	73 (22.8)	247 (77.2)	1	
	II	26 (27.1)	70 (72.9)	1.257 (0.747–2.115)	0.389
	III	5 (62.5)	3 (37.5)	5.639 (1.316–24.161)	0.020
Preoperative Anemia(g/dl)	>10	73 (20.1)	291 (79.9)	1	0.000
	≤10	31 (51.7)	29 (48.3)	4.261 (2.416–7.517)	
Category of surgery	Elective	27 (16.3)	139 (83.7)	1	0.002
	Emergency	77 (29.8)	181 (70.2)	2.190 (1.340–3.578)	
Preoperative Spo_2_ (%)	≥94	78 (20.7)	299 (79.3)	1	0.000
	<94	26 (55.3)	21 (44.7)	4.746 (2.536–8.882)	
Duration of surgery (hr)	≤2	11 (8.8)	114 (91.2)	1	
	2–3	41 (22.3)	143 (77.7)	2.971 (1.462–6.041)	0.003
	>3	52 (45.2)	63 (54.8)	8.554 (4.166–17.566)	0.000
In situ of NG tube	Yes	51 (50.5)	50 (49.5)	5.196 (3.187–8.471)	0.000
	No	53 (16.4)	270 (83.6)	1	
Intra-operative blood loss (22)	≤500	41 (14.6)	240 (85.4)	1	0.000
	>500	63 (44.1)	80 (55.9)	4.610 (2.888–7.358)	
Post-operative albumin (g/dl)	<3.5	68 (56.7)	52 (43.3)	9.045 (5.472–14.950)	0.000
	≥3.5	36 (11.8)	268 (88.2)	1	
Post-operative blood glucose (mmol/L)	≤6.1	33 (36.3)	58 (63.7)	1.891 (1.145–3.122)	0.004
	>6.1	71 (21.3)	262 (78.7)	1	

ASA, American Society of Anesthesiologist; Preoperative Spo_2_, oxy-hemoglobin saturation; NG tube, nasogastric tube; COR, crude odds ratio; CI, confidence interval; AOR, adjusted odds ratio.

In multi-variable logistic regression analysis, variables such as category of surgery, preoperative Spo_2_, duration of surgery, NG tube *in situ*, intra-operative blood loss, and post-operative albumin had a *p*-value of <0.05 and were considered significant associated factors for PPC (Table 4).

**Table 4:** Multi-variable logistic regression analysis of factors associated with post-operative pulmonary complications after thoracic and upper abdominal surgery at comprehensive specialized hospitals in Amhara region, Ethiopia.

		PPC		Odds ratio		
Variables	Category	Yes (*n*, %)	No (*n*, %)	COR (95% CI)	AOR (95% CI)	*P*-value
Age (years)	<65	90 (23.2)	298 (76.8)	1	1	0.377
	≥65	14 (38.9)	22 (61.1)	2.107 (1.036–4.287)	1.531 (0.596–3.933)	
ASA	I	73 (22.8)	247 (77.2)	1	1	
	II	26 (27.1)	70 (72.9)	1.257 (0.747–2.115)	0.841 (0.399–1.770)	0.648
	III	5 (62.5)	3 (37.5)	5.639 (1.316–24.161)	0.883 (0.107–7.317)	0.908
Preoperative Anemia (g/dl)	>10	73 (20.1)	291 (79.9)	1	1	0.199
	≤10	31 (51.7)	29 (48.3)	4.261 (2.416–7.517)	1.653 (0.768–3.559)	
Category of surgery	Elective	27 (16.3)	139 (83.7)	1	1	0.006
	Emergency	77 (29.8)	181 (70.2)	2.190 (1.340–3.578)	2.508 (1.295–4.857)	
Preoperative Spo_2_ (%)	≥94	78 (20.7)	299 (79.3)	1	1	0.002
	<94	26 (55.3)	21 (44.7)	4.746 (2.536–8.882)	3.804 (1.659–8.719)	
Duration of surgery (hr)	≤2	11 (8.8)	114 (91.2)	1	1	
	2–3	41 (22.3)	143 (77.7)	2.971 (1.462–6.041)	2.451 (1.057–5.683)	0.037
	>3	52 (45.2)	63 (54.8)	8.554 (4.166–17.566)	5.239 (2.144–12.803)	< 0.001
In situ of NG tube	Yes	51 (50.5)	50 (49.5)	5.196 (3.187–8.471)	2.878 (1.518–5.458)	0.001
	No	53 (16.4)	270 (83.6)	1	1	
Intraoperative blood loss (ml)	≤500	41 (14.6)	240 (85.4)	1	1	
	>500	63 (44.1)	80 (55.9)	4.610 (2.888–7.358)	2.614 (1.384–4.936)	0.003
Postoperative albumin (g/dl)	<3.5	68 (56.7)	52 (43.3)	9.045 (5.472–14.950)	6.211 (3.382–11.406)	<0.001
	≥3.5	36 (11.8)	268 (88.2)	1	1	
Postoperative blood glucose (mmol/L)	≤6.1	33 (36.3)	58 (63.7)	1.891 (1.145–3.122)	0.826 (0.403–1.693)	0.602
	>6.1	71 (21.3)	262 (78.7)	1	1	

1, reference; ASA, American society of anesthesiologist; Preoperative Spo_2_, oxy-hemoglobin saturation; NG, nasogastric tube;COR, crude odds ratio; CI, confidence interval; AOR, adjusted odds ratio; PPC, postoperative pulmonary complication.

In this study, emergency patients were 2.5 times (AOR: 2.508, 95% CI: 1.295–4.857) more likely to develop PPC than elective patients. Patients with preoperative Spo_2_ < 94% were 3.8 times (AOR: 3.804, 95%CI: 1.659–8.719) more likely to have PPC than patients with preoperative Spo_2_ ≥ 94%. Patients with prolonged surgical duration (>2 h) were 2.5 times more likely to develop PPC than patients whose operations lasted ≤2 h (AOR: 2.451, 95%CI: 1.057–5.683). Patients with an NG tube were 2.9 times (AOR: 2.878, 95%CI: 1.518–5.458) more likely to develop PPC than patients without NG tube *in situ*. Patients with intra-operative blood loss of >500 ml were 2.6 times more likely to develop PPC than patients with blood loss of ≤500 ml (AOR: 2.614, 95%CI: 1.384–4.936). The odds of developing PPC were 6.2 folds (AOR: 6.211, 95%CI: 3.382–11.406) increased in patients with postoperative albumin level <3.5 g/dl compared to patients with postoperative albumin ≥3.5 g/dl (Table 4).

## 4. Discussion

The incidence of post-operative pulmonary complications following thoracic and upper abdominal surgery at comprehensive and specialized hospitals in Amhara region was 24.5% (95% CI: 20.5–28.5). This suggests that post-operative pulmonary complications after thoracic and upper abdominal surgery were still significant.

A study conducted in Albania showed that the incidence of PPC was 27.3% which is in line with our finding (23). In our study, the incidence of postoperative pulmonary complications was higher compared to previous studies in China and Brazil, with the incidence of PPC being 9.7%, 11.5%, and 19%, respectively (24–26). This relatively higher incidence of post-operative pulmonary complications may be due to the grade and complexity of surgery, since all patients in our study underwent major surgery.

The incidence of PPC was lower than in previous studies conducted in Zimbabwe; about 42.4% of the patients who underwent thoracic and abdominal surgeries developed PPC (27). The incidence of PPC in our study was also lower than a study done in Turkey; 58.3% of patients after upper abdominal surgery had PPC (28). This might be due to the higher percentage of ASA-class I and younger patients in our study.

In our study, different types of pulmonary complications were identified in the post-operative period. Among those complications, pneumonia was the most common, followed by respiratory infection and respiratory failure, which is similar to other previous studies (24, 28–30).

The results of our study demonstrated that the incidence of post-operative pulmonary complications after thoracic and upper abdominal surgery was significantly associated with emergency procedures, preoperative Spo_2_ < 94%, prolonged duration of surgery >2 h, patients with an NG tube, intra-operative blood loss >500 ml and a postoperative albumin level <3.5 g/dl. Clinical measures targeted at these factors are essential to prevent the development of PPC.

Our study demonstrated that emergency patients were more than twice as likely to develop PPC than elective patients, and it was one of the main factors for postoperative pulmonary complications, which is consistent with other studies (17, 31, 32). This may be due to the urgent nature of the procedure, the clinical characteristics of patients, as well as preoperative diagnoses and surgical strategies adopted in emergency situations, which carry higher risks for PPC due to impaired bowel motility and obstructive bowel lesions predisposing to PPC (33).

Patients with preoperative Spo_2_ < 94% were more likely to develop PPC than patients with preoperative Spo_2_ ≥ 94% and this finding is in agreement with similar studies (24, 31, 34). A longer duration of surgery was associated with PPC. The reasons may be that the prolonged operation duration means prolonged anesthesia time that depresses movements of inter-costal muscles, alters the shape and motion of the chest wall, and diminishes ribcage excursion, affecting lung mechanics and resulting in a decrease in functional residual capacity (35). In our study, patients with prolonged surgical times were more likely to develop PPC than those whose surgeries lasted less than 2 h, which was consistent with previous studies (27, 31, 34).

Patients with an NG tube were more likely to develop PPC than patients without an NG tube, which was in agreement with previous studies (16, 36, 37). The exact mechanism of how NG tube usage is associated with PPC is not clear, but interference with cough due to discomfort, incomplete closure of the glottis, fostering transfer of microorganisms from the oro-pharynx to the airways, and favoring silent aspiration are possible mechanisms (38).

In our study, patients with intra-operative blood loss of >500 ml were more likely to develop PPC than patients with blood loss of less than 500 ml which was in line with previous studies (12, 30). Excessive blood loss during surgical procedures calls for judicious replacement to restore a normovolemic state. However, infusion of large volumes of fluids intra-operatively is associated with a higher incidence of PPC (39).

Our study revealed that the risk of developing PPC was higher in patients with a postoperative albumin level less than 3.5 g/dl compared to those with a postoperative albumin level greater than 3.5 g/dl. This finding was in agreement with other studies (24, 34). This could be due to a low blood protein level, which results in vascular endothelial dysfunction, or by reducing colloid osmotic pressure. It contributes to PPC by decreasing the oncotic forces that favor the retention of fluid in the microvasculature (40).

## 5. Limitation of the study

This study has certain shortcomings that must be considered. First, we have not performed follow-up after seven days of the postoperative period. The long-term risk of PPC after surgery in this study remains unclear. Furthermore, the attending physician was in charge of the diagnosis of PPCs; thus, potential estimation bias for actual PPC incidence was unavoidable.

## 6. Conclusion and recommendation

The incidence of post-operative pulmonary complications after thoracic and upper abdominal surgery was a common postoperative morbidity. The most common complication was pneumonia, followed by respiratory infection and respiratory failure. Pre-operative SpO_2_ of less than 94%, duration of surgery more than 2 h, patients with NG tubes, intra-operative blood loss greater than 500 ml, and post-operative albumin less than 3.5 g/dl were factors associated with post-operative pulmonary complications.

Surgical team members should place more emphasis on detecting and reducing modifiable risk factors during the peri-operative period, and close post-operative follow-up is important for a better outcome for the patient after thoracic and upper abdominal operations.

## Data Availability

The original contributions presented in the study are included in the article, further inquiries can be directed to the corresponding author.
